# Construction and Validation of a Nomogram Model for Stress Urinary Incontinence of Late Pregnancy

**DOI:** 10.1002/nop2.70702

**Published:** 2026-07-31

**Authors:** Shanshan Shan, Ying Chen, Junying Li, Xin Zhang, Yu Chen, Hui Jiang, Yaoxiang Duan

**Affiliations:** ^1^ Delivery Room Shanghai First Maternity and Infant Hospital Shanghai China; ^2^ Outpatient Department Shanghai First Maternity and Infant Hospital Shanghai China; ^3^ Nursing Department Shanghai First Maternity and Infant Hospital Shanghai China

**Keywords:** nomogram, risk prediction, stress urinary incontinence, third trimester of pregnancy

## Abstract

**Objective:**

To establish and validate a nomogram model for stress urinary incontinence of late pregnancy and then evaluate its effectiveness.

**Design:**

Single‐center cross‐sectional study.

**Methods:**

From January 2025 to June 2025, 414 pregnant women in the late stage of pregnancy who established medical records at a tertiary obstetrics and gynaecology hospital in Shanghai were surveyed through questionnaire. The patients were divided into the modelling group and the validation group in a ratio of 7:3. Among them, 290 cases were used for the training set and 124 cases for the validation set. The data were collected and verified by two individuals, and then a single‐factor analysis was conducted. The statistically significant variables in the training set were subjected to Lasso regression analysis to further screen out potential influential variables. Subsequently, a multivariate Logistic regression analysis was performed. Finally, six variables were included in the model. A nomogram model was established and then validated using receiver operating characteristic (ROC) curves, calibration curves, Hosmer‐Lemeshow goodness of fit test, and decision curve analysis.

**Results:**

Six factors were included in the model, including age, history of miscarriage, pelvic floor muscle training, family history of urinary incontinence, smoking habits and coffee consumption. The AUC of the training set was 0.845 (95% CI 0.799–0.891), with specificity and sensitivity being 0.729 and 0.849 respectively, and the optimal cut‐off value was 0.505. The area under the curve of the validation set was 0.805 (95% CI 0.725–0.885), with specificity and sensitivity being 0.636 and 0.870 respectively, and the optimal cut‐off value was 0.469. Calibration tests and DCA demonstrated favourable discrimination and clinical practicability of the nomogram.

**No Patient or Public Contribution:**

No patient or public representatives were involved in the design, statistical analysis or manuscript writing procedures of this research. But the established nomogram avoids complex statistical calculation, enables obstetric midwives to rapidly identify high‐risk women at the prenatal clinic, and facilitates targeted pelvic floor muscle training health education and lifestyle intervention to reduce gestational SUI burden for pregnant populations.

## 1 Introduction

The International Continence Society (ICS) defines urinary incontinence (UI) as the involuntary leakage of urine (Abrams et al. 2003). It is one of the most common pelvic floor dysfunction disorders, resulting from defects or injuries to the pelvic floor support tissues. Due to the unique pelvic floor structure and hormone levels in women, the incidence of UI in women is much higher than that in men. UI has become a widespread cross‐cultural global health issue, and the World Health Organization has listed it as one of the five chronic diseases threatening women's health (Minassian et al. 2003). Although UI is not a fatal disease, it has adverse effects on women's social relationships and mental and physical health, causing them to worry about incontinence and reduce social activities, leading to depression or anxiety; it also affects sexual function and marital relationships (Kelly and Vichayavilas 2009).

Stress Urinary Incontinence is the most common type of UI during pregnancy and postpartum, accounting for over 80% of UI in pregnant and postpartum women. SUI refers to the involuntary leakage of urine caused by sudden increases in abdominal pressure, rather than by detrusor muscle contractions or the tension pressure of the bladder wall on urine. Stress urinary incontinence in women is a multifactorial disease, and one of the important risk factors is the trauma during pregnancy and childbirth (Moossdorff‐Steinhauser et al. 2021). As pregnancy progresses, the uterus gradually enlarges, increasing the pressure on the female pelvic floor, which may lead to stretching of the pelvic floor muscles and ligaments, making it more prone to damage. Relevant studies have shown that women with urinary leakage symptoms during pregnancy have more than twice the risk of experiencing urinary leakage again within 15 years after giving birth compared to those without such symptoms (Dolan et al. 2003). Results from a meta‐analysis showed that urinary incontinence in pregnancy significantly increases the risk of urinary incontinence 2–18 months postpartum, with an OR of 5.32 (95% CI 4.13–6.86) (Hage‐Fransen et al. 2021). Therefore, early prevention and management of SUI should be initiated during the prenatal period.

Studies have shown that the incidence of UI is high during pregnancy but decreases after childbirth. The ICS reports that the incidence of UI is approximately 7%–39% (Linde et al. 2019). Large‐scale surveys abroad have found that the incidence of UI gradually increases from before pregnancy to during pregnancy, reaching a peak in the third trimester. A systematic review indicates that based on 44 studies involving 88,305 women, the average incidence rate was 41.0%, the incidence of urinary incontinence during the first trimester, the second trimester, and the third trimester of pregnancy is 9%, 19%, and 34% respectively (Moossdorff‐Steinhauser et al. 2021). Compared with Europe, the prevalence of UI during pregnancy in China is slightly lower. A study by Zhong (Zhong et al. 2026) involving 426 nulliparous women found that the incidence of urinary incontinence during pregnancy was 27.7%, which decreased to 20.2%, 16.2% and 14.3% at 42 days postpartum, 16.2% at 3 months postpartum, and 14.3% at 6 months postpartum.

Existing studies have identified several risk factors for gestational SUI, including advanced age, constipation, coffee consumption and smoking exposure, dietary patterns are also associated with intestinal peristalsis and constipation status (Martins et al. 2010; Li et al. 2023; Wang et al. 2022; Soave et al. 2019). Studies have shown that regular pelvic floor muscle exercises can prevent and control the occurrence of SUI during pregnancy (Wu et al. 2025). However, most perinatal women have insufficient awareness of stress urinary incontinence, or perhaps due to the embarrassment associated with the disease, they rarely seek consultation or treatment for this issue (Cooke et al. 2018). Routine universal pelvic floor muscle intervention for all pregnant women also increases unnecessary nursing workload. At present, few studies have constructed a targeted prediction model for gestational SUI to screen high‐risk populations efficiently.

Given the above research gaps, this study aimed to construct and validate a nomogram model for predicting SUI in late pregnancy based on multiple risk factors. This model is expected to facilitate rapid risk stratification in obstetric outpatient nursing, guide targeted pelvic floor health education and intervention, reduce the incidence of SUI, and improve the quality of life of pregnant women.

## 2 Materials and Methods

### 2.1 Research Subjects

This was a single‐center cross‐sectional study. The research objects are pregnant women who established medical records at a tertiary obstetrics and gynaecology hospital in Shanghai from January 2025 to June 2025. All participants completed questionnaires during their routine prenatal examination at 28~30 weeks of gestation. The study aims to summarise and analyse the possible risk factors related to stress urinary incontinence (SUI) during pregnancy. Inclusion criteria: ① Age ≥ 18 years; ② Diagnosed as being in a pregnant state; ③ Those who have registered at our hospital, have regular prenatal check‐ups and plan to give birth at our hospital; ④ Those with clear consciousness, who have given informed consent, are willing to participate in this research project and no loss to follow‐up. Exclusion criteria: ① History of urinary incontinence before pregnancy; ② History of abdominal or vaginal surgeries; ③ Previous history of urinary tract infection, kidney disease, and pelvic surgeries; ④ Individuals with other factors that may cause urinary disorders (spinal cord lesions, diabetes insipidus, etc.); ⑤ Placenta previa, threatened premature labour, abnormal amniotic fluid volume, fetal growth restriction, vaginal bleeding, etc.; ⑥ Individuals with significant physical defects and major diseases; ⑦ Individuals with visual or auditory impairments as they can't fill out questionnaire independently; ⑧ Individuals under the age of 18.

### 2.2 Calculation of Sample Size

The Events Per Variable (EPV) rule was adopted for sample size calculation, which required the sample size to be at least 10 times the number of independent variables (Chen 2018). In this study, the dependent variable is binary (whether SUI occurs or not), and the preliminary estimated meaningful independent variables are 10. Therefore, the sample size of the case group in this study is approximately 100 cases. According to the literature, the incidence of SUI during pregnancy in China is approximately 26.7%, so the sample size required for Logistic regression modelling in this study is at least 100 ÷ 26.7% = 375 cases. We surveyed a total of 420 pregnant women. Among them, 3 were lost to follow‐up due to visiting the outpatient clinic during the filling process, and 3 refused to submit the forms for personal reasons, and the final valid sample included 414 cases. All eligible participants were randomly divided into a modelling group (*n* = 290) and an internal validation group (*n* = 124) at a 7:3 ratio.

### 2.3 Methods

#### 2.3.1 Research Tools

① General information questionnaire: Collected data on age, pre‐pregnancy body mass index (BMI), educational level, occupation, family economic status, and number of pregnancies.

② Risk factors assessment form: Previous childbirth history, history of miscarriage, constipation, exercise habits, pelvic floor muscle training, family history of UI, smoking habits, coffee consumption, and daily diet.

③ Urinary Incontinence Assessment Tool: In this study, the International Consultation on Incontinence Questionnaire short form (ICI‐Q‐SF) (Huang et al. 2008), which is internationally recognised, was used as the measurement tool. The score range is from 0 to 21, with 0 indicating no urinary incontinence, 1~7 indicating mild, 8~13 indicating moderate, and 14~21 indicating severe. This questionnaire consists of 4 items, namely the frequency of urine leakage, the volume of urine leakage, and the impact of urine leakage on the patient, as well as questions related to the type of urinary incontinence. Among the last group of 8 questions related to the type of UI, only those pregnant women who answered at least one of the following questions could be considered to meet the clinical diagnosis of SUI: “leaking urine during coughing or sneezing”, “leaking urine during physical activities”, “leaking urine during physical exercise”, with no “leaking urine before reaching the bathroom”. The internal consistency coefficient of this questionnaire in the Chinese population is 0.71, indicating moderate internal consistency (Huang et al. 2008).

#### 2.3.2 Data Collection Methods

All the data were collected one‐on‐one by the members of the research team who had received unified training and they were blinded to participants' baseline influencing factors to reduce assessment bias. Pregnant women who met the inclusion and exclusion criteria were selected. After obtaining informed consent, the team members used a unified guideline to explain in detail the precautions for filling out the questionnaire to the research subjects. During the prenatal check‐ups, general information questionnaires, risk factor assessment forms, and the International Consultation on Incontinence Questionnaire—Short Form (ICIQ‐SF) were administered to the pregnant women. After the questionnaires were completed, they were collected by the team members on the spot. The team members also checked for any omissions or errors in the questionnaires.

#### 2.3.3 Statistical Analysis

The analysis was conducted using SPSS 23.0 statistical software. For the measurement data that followed a normal distribution, the mean ± standard deviation (*x* ± *s*) was used to represent them, and the *t*‐test was employed for comparisons between groups; for the data that follows non‐normal distribution, the median (*M*) and interquartile range (*Q*) were used for representation, and the Mann–Whitney *U* test was used for comparisons between groups. The count data were expressed as the number of cases, and the Chi‐square test was used for comparisons between groups, with Fisher's exact test used when the sample size was less than 40. Variables with *p* < 0.05 in univariate analysis were included in subsequent Lasso regression and multivariate binary Logistic regression. The nomogram was drawn using the R 4.3.3 software, and bootstrap resampling was conducted 1000 times to draw calibration curves for internal verification of the model. The model was evaluated using Hosmer‐Lemeshow goodness‐of‐fit, calibration curves, and receiver operating characteristic (ROC) curves. No obvious missing data was found in valid questionnaire.

#### 2.3.4 Ethical Considerations

The study has been approved by the Ethics Committee of the Shanghai First Maternity and Infant Hospital (Ethical Number KS2402).

## 3 Results

### 3.1 Univariate Analysis of SUI During Pregnancy

By comparing the clinical data of the two groups using single‐factor analysis, age, educational level, gravidity and parity history, history of miscarriage, constipation, pelvic floor muscle training, family history of urinary incontinence, smoking habits, coffee consumption, breakfast type and staple food structure were all influencing factors (*p* < 0.05), as seen in Table 1.

**TABLE 1:** Comparison of the clinical data between the two groups of patients (*n* = 290).

Item	Non‐SUI (*n* = 118)	SUI (*n* = 172)	*χ* ^2^/*Z*	*p*
Age [year, Median (P25, P75)]	29.00 (28.00, 32.00)	32 (29.75, 35.00)	6064	< 0.001
Pre‐pregnancy BMI [kg/m^2^, Median (P25, P75)]	21.71 (20.33, 24.0)	22.35 (20.08, 25.06)	9519.5	0.371
Education level			14.575	0.002
High school and below	2	9		
Junior college	24	11		
Undergraduate	45	73		
Master's degree or above	47	79		
Occupation type			0.357	0.550
Office work mainly	100	150		
Physical activities mainly	18	22		
Financial situation (Yuna, year)			5.034	0.169
≤ 20	36	46		
20–40	54	64		
40–60	12	27		
≥ 60	16	35		
Number of pregnancies			8.266	0.016
1	90	117		
2	24	32		
≥ 3	4	23		
Previous childbirth history			9.377	0.025
No	92	132		
History of caesarean section	2	5		
History of vaginal delivery	13	31		
Others	11	4		
History of miscarriage			13.224	< 0.001
No	102	115		
Yes	16	57		
Vomiting during pregnancy			1.000	0.317
No	77	101		
Yes	41	71		
Gestational weeks of early pregnancy symptoms [week, Median (P25, P75)]	6 (6, 7)	6 (6, 7)	10,104	0.949
Constipation during pregnancy			6.087	0.048
No	59	68		
Occasionally	34	45		
Frequently	25	59		
Exercise habits			0.135	0.935
Rarely	35	50		
Occasionally (1~2 times per week, at least half an hour each time, with a heart rate of at least 100 beats per minute)	49	75		
Frequently (at least 3 times a week, for at least half an hour each time, with a heart rate of at least 100 beats per minute)	34	47		
Pelvic floor muscle training			5.097	0.024
Yes	64	117		
No	54	55		
Family history of urinary incontinence			37.249	< 0.001
No	74	47		
Yes	35	109		
Uncertain	9	16		
Smoking habits in a year			9.853	0.002
Yes	0	0		
No, but will be exposed to second‐hand smoke	4	27		
No	114	145		
Coffee consumption in a year			16.305	< 0.001
Frequently (at least 3 times per week)	8	38		
Occasional (1~2 times per week)	28	50		
Rarely	82	84		
Breakfast type			4.378	0.036
Cookies, bread, milk beverages	78	134		
Porridge, pancakes, buns, milk, eggs	40	38		
Lunch or dinner			1.274	0.259
Fast food in the or take‐away	42	79		
Cooking at home	99	113		
Habit of midnight snack			1.797	0.616
Never	78	120		
Rarely (1 time per week)	36	29		
Occasionally (2~3 times per week)	24	30		
Frequently (4 times per week and above)	3	13		
Composition of staple food			34.912	< 0.001
Mainly rice and wheat flour, with a small amount of coarse grains and tubers	54	115		
Rice, white flour, coarse grains and tubers in equal quantities	40	12		
Mainly coarse grains and tubers, with a small amount of rice and white flour	20	35		
Rice and white flour only	4	10		

### 3.2 Lasso Regression Screening Results

**FIGURE 1:**
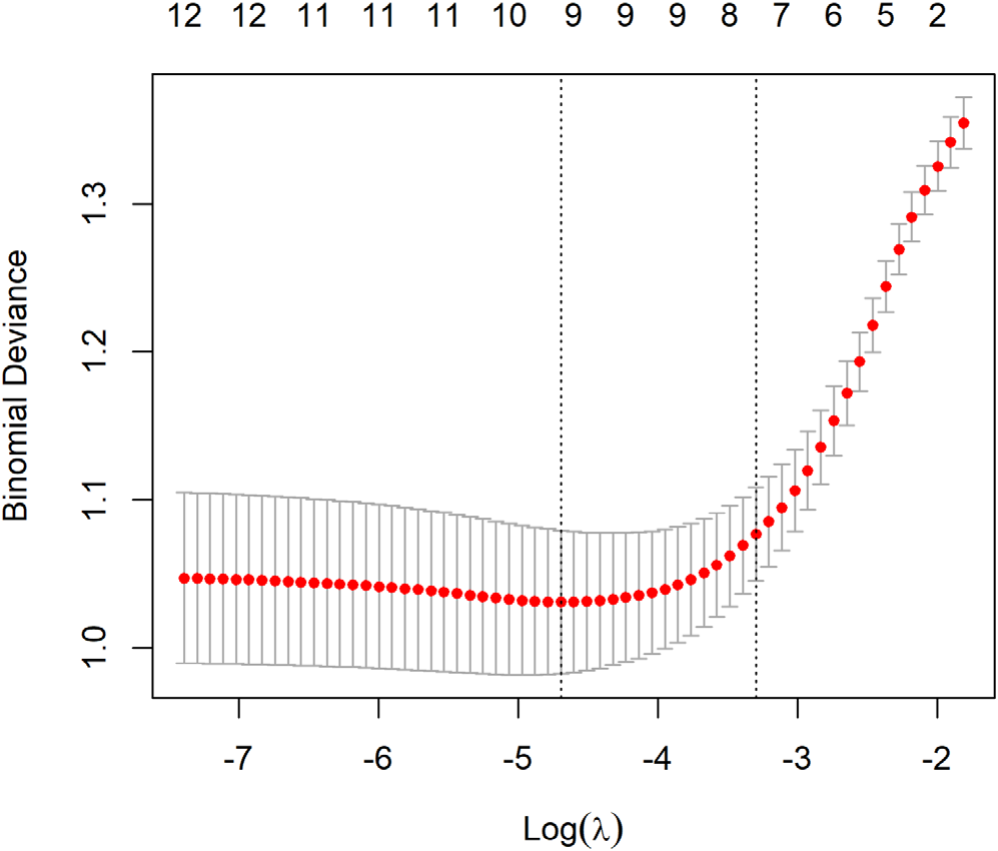
Cross‐validation results of Lasso regression.

**FIGURE 2:**
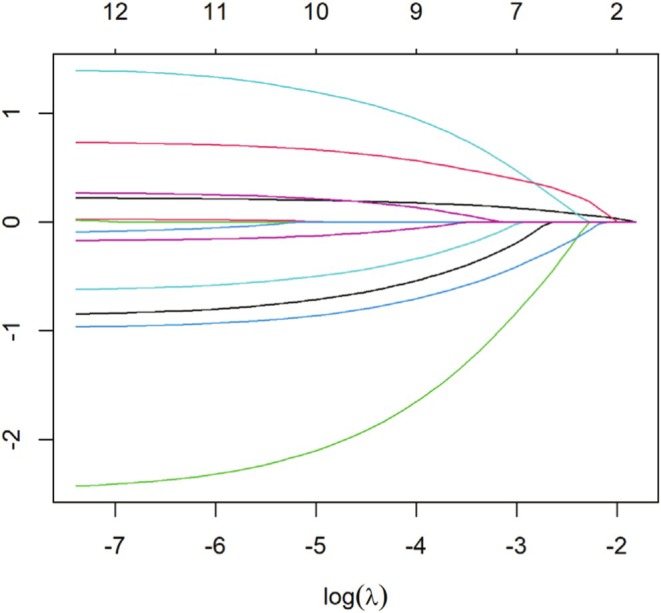
Coefficient paths of Lasso regression.

To avoid the model overfitting in the training set, Lasso regression was used to screen predictive variables. Firstly, all variables with *p* < 0.05 in univariate analysis were enrolled as candidate predictors. Then, 10‐fold cross‐validation was applied to determine the optimal *λ* value (*λ*.lse = 0.037) to minimise prediction error. Variables with non‐zero regression coefficients under the optimal *λ* were retained for subsequent multivariate logistic regression. Ultimately, 8 variables were retained: age, history of miscarriage, constipation, pelvic floor muscle training, family history of UI, smoking habits, coffee consumption and breakfast type. As is shown in Figures 1 and 2.

### 3.3 Multifactor Logistic Regression Analysis of SUI in Pregnant Women

Using the R 4.3.3 software, 8 variables selected by lasso regression were subjected to multivariate Logistic regression analysis to construct a prediction model. The assignment of independent variables is shown in Table 2. The results demonstrated that age, history of miscarriage, pelvic floor muscle training, family history of UI, smoking habits and coffee consumption were independent risk factors affecting SUI (*p* < 0.05). The result is shown in Table 3.

**TABLE 2:** Assignment table of independent variables.

Variable	Assignment
History of miscarriage	No = 1, Yes = 2
Constipation during pregnancy	No = 1, Occasionally = 2, Frequently = 3
Pelvic floor muscle training	No = 1, Yes = 2
Family history of urinary incontinence	No = 1, Yes = 2, Uncertain = 3
Smoking habits in a year	Yes = 1, No, but will be exposed to second‐hand smoke = 2, No = 3
Coffee consumption in a year	Frequently (at least 3 times per week) = 1, Occasional (1 ~ 2 times per week) = 2, Rarely = 3
Breakfast type	Cookies, bread, milk beverages = 1, Porridge, pancakes, buns, milk, eggs = 2

**TABLE 3:** Binary logistic regression analysis of factors affecting SUI during pregnancy.

Independent variable	*B*	SE	Wald *χ* ^2^	*p*	OR (95% CI)
Age	0.208	0.049	17.604	< 0.001	1.231 (1.117–1.356)
History of miscarriage (Ref = No)	1.341	0.390	11.831	0.001	3.824 (1.781–8.211)
Constipation during pregnancy (Ref = No)			2.595	0.273	
Occasionally	0.030	0.375	0.006	0.937	1.030 (0.494–2.147)
Frequently	0.578	0.382	2.286	0.131	1.782 (0.843–3.768)
Pelvic floor muscle training (Ref = No)	−0.768	0.333	5.310	0.021	0.464 (0.241–0.892)
Family history of urinary incontinence (Ref = No)			13.514	0.001	
Yes	1.219	0.332	13.489	< 0.001	3.383 (1.765–6.484)
Uncertain	0.764	0.567	1.831	0.178	2.147 (0.706–6.528)
Smoking habits (Ref = No, but will be exposed to second‐hand smoke)	−2.470	0.637	15.029	< 0.001	0.085 (0.024–0.295)
Coffee consumption (Ref = Frequently)			18.444	< 0.001	
Occasional (1~2 times per week)	−0.701	0.545	1.653	0.199	0.496 (0.170–1.445)
Rarely	−1.825	0.502	13.209	< 0.001	0.161 (0.060–0.431)
Breakfast type (Ref = Cookies, bread, milk beverages)	−0.580	0.373	2.421	0.120	0.560 (0.270–1.162)
Constant	−3.198	1.554	4.237	0.040	—

### 3.4 Construction of a Nomogram Model for SUI in Pregnant Women

**FIGURE 3:**
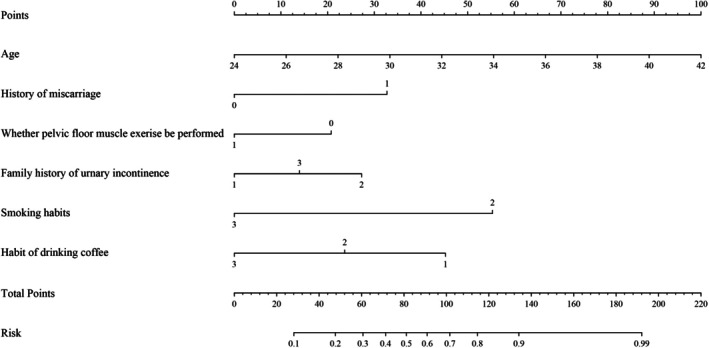
Nomogram model of SUI of pregnant women.

Based on multi‐factor Logistic regression analysis, the screened 6 predictive factors were included in the model to construct a nomogram model for SUI. The total score ranges from 0 to 220; the higher the total score, the higher the risk value of SUI, as seen in Figure 3.

### 3.5 Evaluation of the Nomogram Model for SUI in Pregnant Women

**FIGURE 4:**
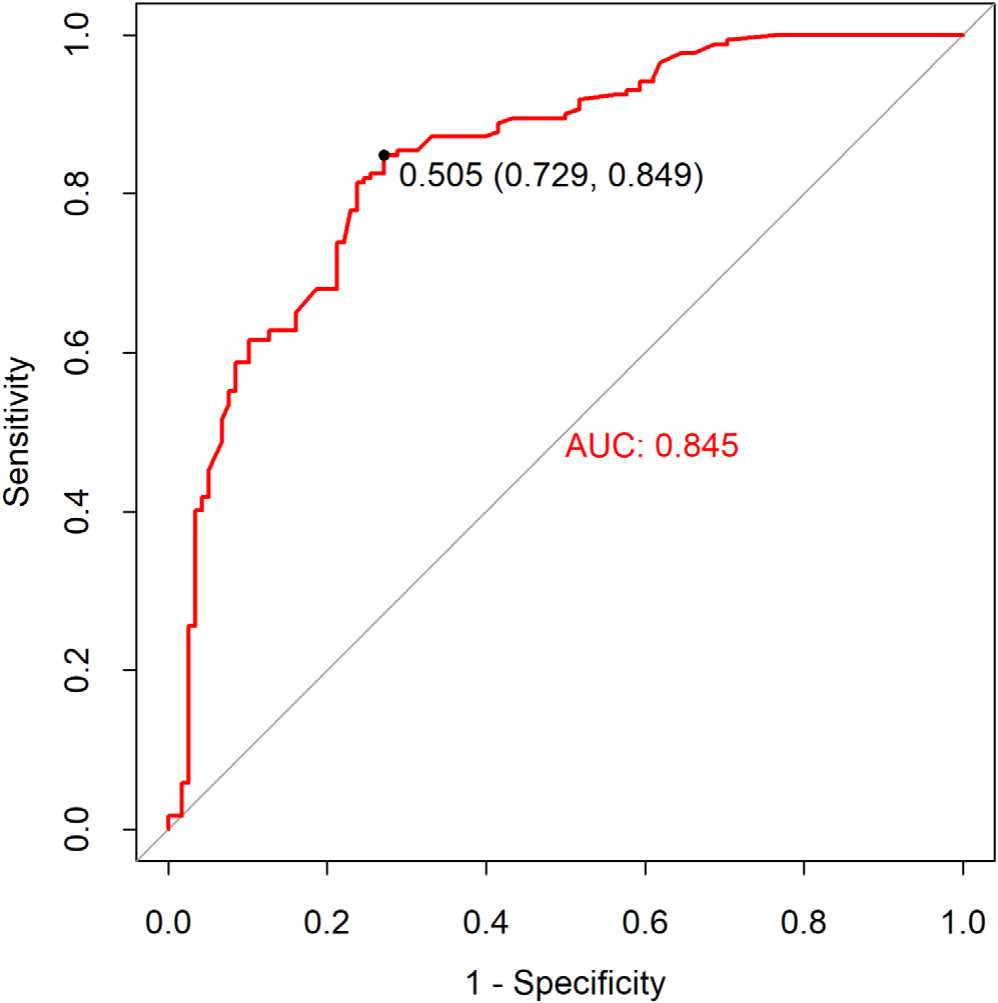
ROC curve of the nomogram model (training set).

**FIGURE 5:**
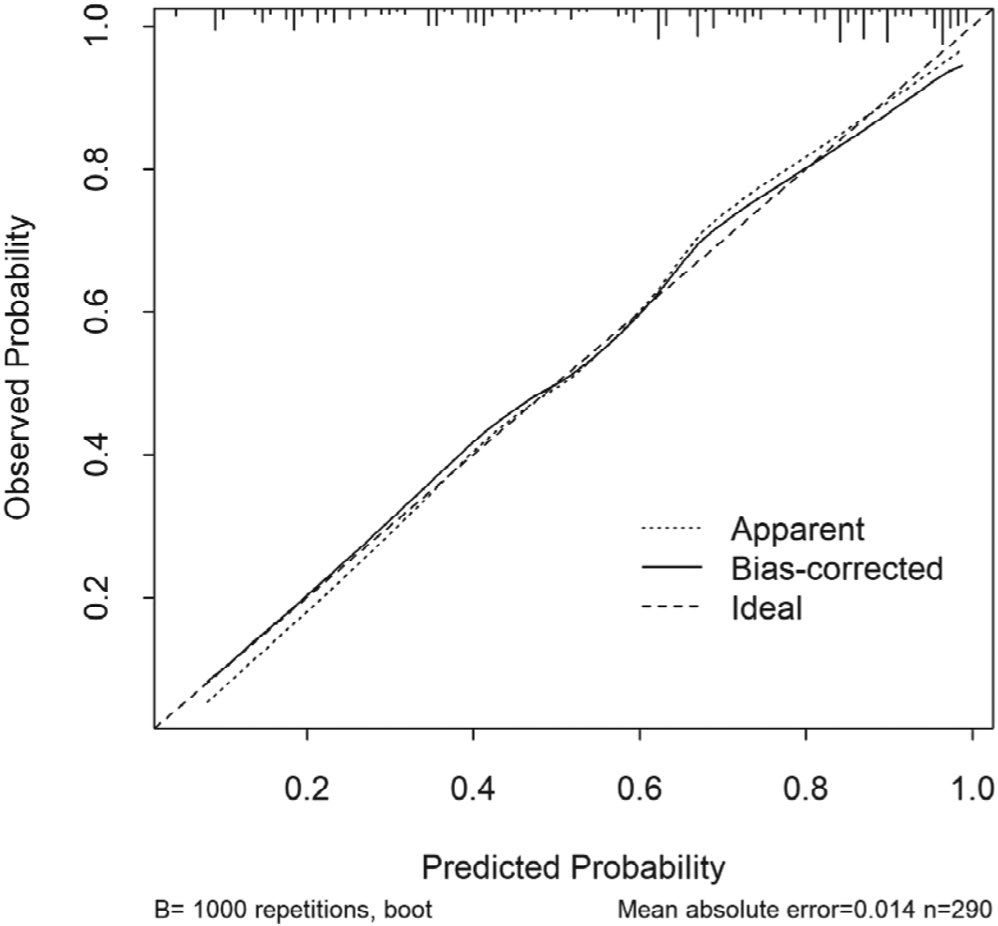
Hosmer‐Lemeshow fit results of the prediction model (training Set).

Drawing ROC curves to evaluate the discriminability of the model, the AUC of the training set is 0.845 (95% CI 0.799–0.891), with a sensitivity of 0.849 and a specificity of 0.729. The optimal cut‐off value was 0.505. The results are shown in Figure 4. The calibration accuracy of the model was evaluated through the Hosmer‐Lemeshow goodness‐of‐fit test and calibration curve (Bootstrap method, *n* = 1000). The results showed that the *p*‐value of the training set was 0.16, the average absolute error was 0.015, and the consistency index was 0.827, as shown in Figure 5.

### 3.6 Internal Validation of the Nomogram Model for SUI in Pregnant Women

**FIGURE 6:**
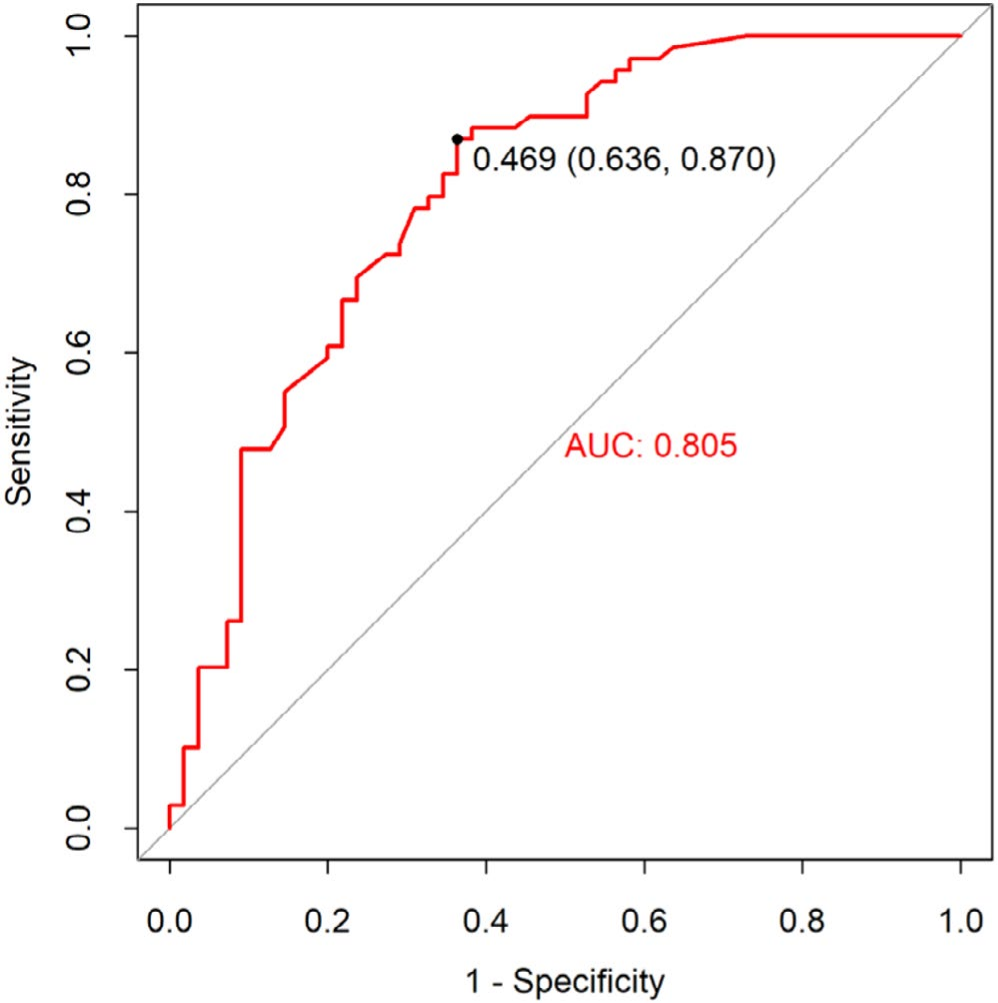
ROC curve of the nomogram model (validation set).

**FIGURE 7:**
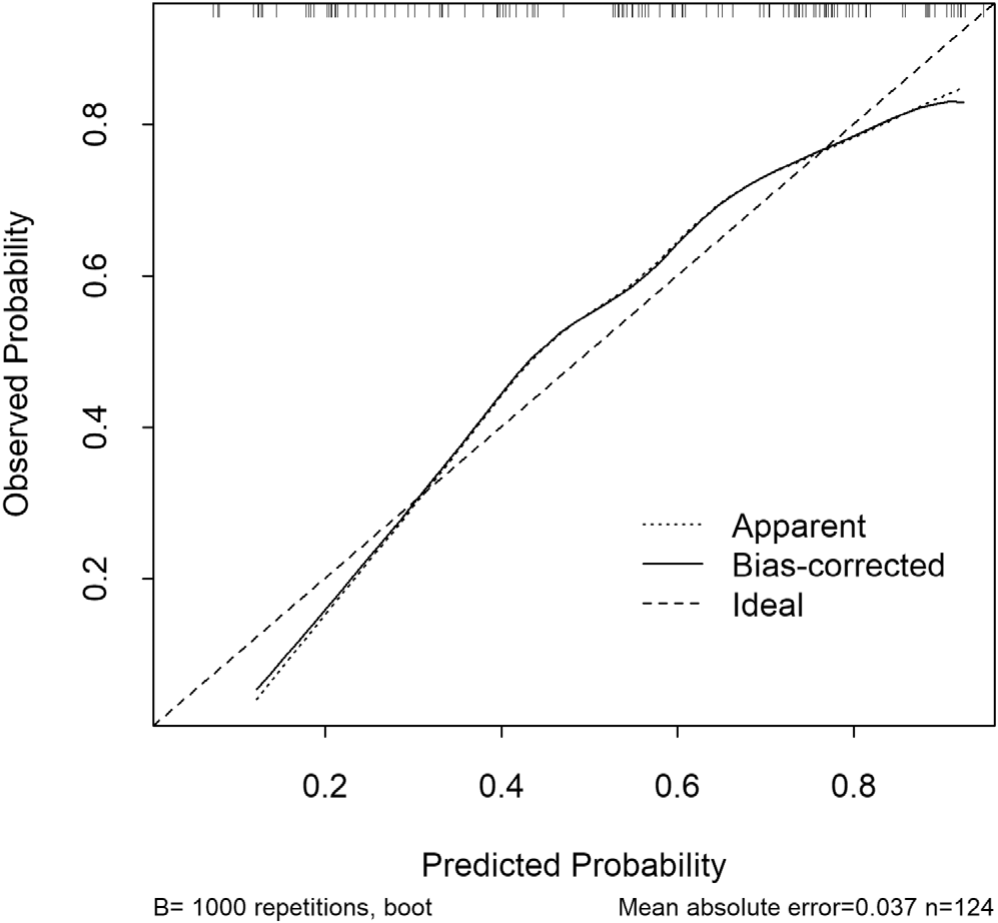
Hosmer‐Lemeshow fit results of the prediction model (validation set).

ROC curve analysis results showed that the AUC of the nomogram model predicting SUI in internally validated was 0.805 (95% CI 0.725–0.885), with a specificity of 0.636 and a sensitivity of 0.870. The optimal cut‐off value was 0.469. The results are shown in Figure 6. The *p*‐value of the validation set is 0.65, which is greater than 0.05. The mean absolute error is 0.035 and the concordance index is 0.804. This indicates that the performance of the model is approaching the ideal predictive performance. As shown in Figure 7.

### 3.7 Clinical Utility Test of the Training and Validation Set Models

**FIGURE 8:**
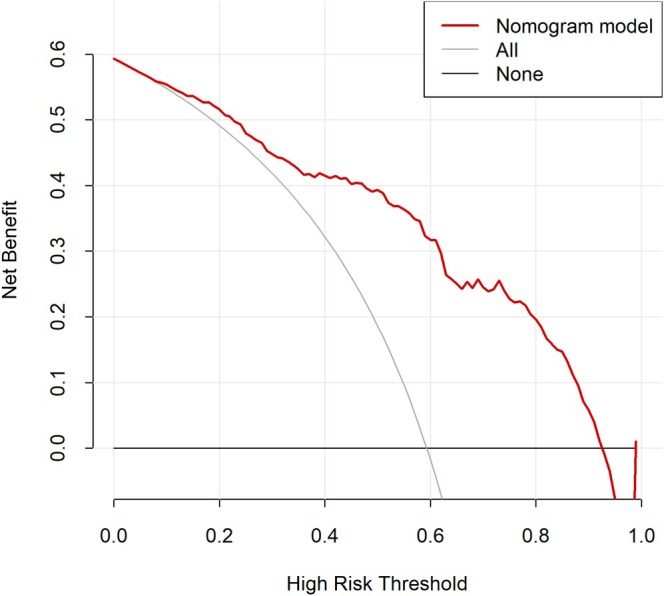
Decision curve of SUI during pregnancy (training set).

**FIGURE 9:**
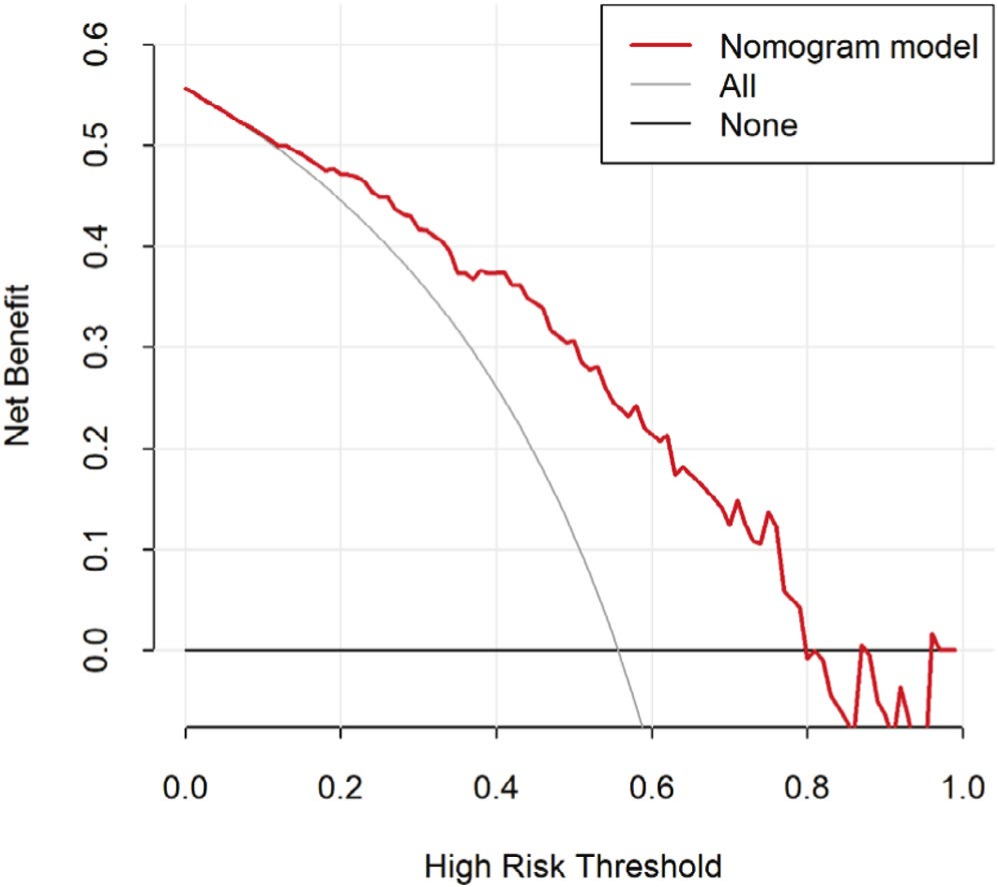
Decision curve of SUI during pregnancy (validation set).

As shown in Figure 8, the training set model predicted the probability of SUI occurrence within the range of 1%–92%. The validation set model predicted the probability of SUI occurrence within the range of 1% to 81%, as in shown in Figure 9. Indicating a significant application potential in clinical practice.

## 4 Discussion

### 4.1 Influencing Factors

This study constructed a nomogram model containing six independent influencing factors for SUI in late pregnancy. Both the modelling group and validation group achieved high AUC values, indicating that the model has excellent discriminative ability and calibration.

Stress urinary incontinence during pregnancy is often considered closely related to the mother's age. As age increases, the contractility of the pelvic floor muscle fibres decreases, which may lead to a decline in the stability of the pelvic floor structure (Dai et al. 2023). Given the rising proportion of women of advanced maternal age and those with multiple gestations undergoing pregnancy in China, these subgroups warrant targeted clinical surveillance and care.

Pelvic floor muscle training is a protective factor for stress urinary incontinence during pregnancy (OR = 0.464, 95% CI 0.241–0.892). Soave's (Soave et al. 2019) research indicates that pelvic floor muscle training, which involves repetitive voluntary contractions of the pelvic muscles, can effectively control urinary leakage and has the potential to prevent and treat urinary incontinence during pregnancy to a certain extent. The possible reason is that through the contraction training of the pelvic floor muscles, the pelvic floor can be stabilised, raising the urethra, vagina, and rectum and resisting downward movement. Although there are no specific regulations on the time and frequency of pelvic floor muscle training, experts believe that it must be conducted under the guidance and supervision of professionals because the techniques for verbal guidance are difficult to master and compliance is poor (Ghaderi et al. 2022).

Lifestyle changes are important influencing factors for urinary incontinence. It is worth noting that in our study, there were no pregnant women who smoked, but the vast majority of pregnant women were exposed to second‐hand smoke. In this study, the risk of SUI in pregnant women exposed to second‐hand smoke increased by 11.7 times, underscoring the necessity of targeted health education to encourage pregnant women to avoid second‐hand smoking throughout gestation. Consistently, Fernanda (Caruso et al. 2020) conducted a survey on pregnant women at 12~20 weeks of gestation and demonstrated that the risk of SUI was eight times that of non‐smokers. The possible explanation for smoking associated with SUI is smoking associated cough, antiestrogenic effect on collagen of urethra and bladder neck, and decreased smooth muscle tone (Bump and McClish 1992). Caffeine overconsumption via coffee or strong tea serves as a modifiable dietary risk factor for gestational SUI. Our research has confirmed that coffee consumption during pregnancy is a risk factor for stress urinary incontinence (SUI). The possible explanation for this is that high caffeine intake, through overactive bladder (OAB) as an intermediate variable, indirectly increases the risk of SUI. Wang et al. (2022) demonstrated that excessive caffeine intake elevated the odds of overactive bladder (OAB) by 1.763‐fold in pregnant women, consequently increasing the incidence of UI. Incorporating smoking and coffee into the nomogram can further screen out high‐risk groups for SUI.

### 4.2 Impact on Nursing Practice

This nomogram is highly applicable to routine obstetric nursing work. At present, most hospitals lack targeted SUI risk screening tools for pregnant women, and many women refuse to consult medical staff due to shyness about urinary symptoms. Combined with the optimal cut‐off value of the validation group (0.469), nurses can use the nomogram to complete rapid risk stratification during prenatal examination at 28~30 weeks of gestation.

For low‐risk pregnant women, nurses can carry out routine pelvic floor health education and popularise basic knowledge of gestational SUI. For high‐risk groups, one‐to‐one professional guidance on standardised pelvic floor muscle training is required. Meanwhile, targeted health education should be provided to advise pregnant women to avoid long‐term second‐hand smoke exposure and excessive coffee intake. Hospitals can also compile illustrated health brochures and launch short‐video popular science to eliminate pregnant women's psychological barriers and improve treatment compliance.

Our hospital has established a midwifery consultation service after the 28th week of pregnancy, providing pregnant women with health consultations during pregnancy and guidance related to childbirth, which also ensures that every pregnant woman receives consultations and guidance related to urinary incontinence in a private consultation room. The establishment of this model can be used for initial screening in the outpatient clinic, completing the risk stratification of urinary incontinence and conducting preliminary intervention for high‐risk groups. When a pregnant woman visits for the first time, the midwife will score each of the 6 indicators in the nomogram one by one, and after accumulating the total score, read the SUI prediction probability against the scale. When the predicted probability is ≥ 0.469, a high‐risk group for SUI is considered. In the following period, our midwives will conduct routine follow‐up for low‐risk groups and provide one‐on‐one guidance on pelvic floor muscle training for high‐risk groups. At the same time, we have also produced health education handbooks, including a correct understanding of SUI, pregnancy smoking cessation, reducing caffeine intake, pelvic floor muscle training, so that pregnant women can correctly understand and actively cooperate with the treatment of urinary incontinence, and improve the psychological tendency of avoiding medical treatment when seeking medical care. This model can be integrated into the standardised workflow of midwifery consultation. It helps nursing staff implement hierarchical and individualised interventions, reduce the overall incidence of SUI, and avoid the waste of nursing resources caused by universal intervention for all pregnant women. Unfortunately, we have not yet established a systematic follow‐up system, so the effect of the intervention hasn't been determined.

### 4.3 Limitations

Our research has some limitations. Firstly, the sample size was calculated using the EPV method, which is a rough calculation method and may have the problem of failing to take into account the collinearity of variables.

Secondly, all the data were derived from self‐administered questionnaires, which may lead to recall bias and reporting bias. We aim to develop an assessment model that can be quickly screened by midwives. Therefore, factors such as socioeconomic status, nutritional status, and obstetric complications, which may also affect the final results, were not included. Additionally, the internal consistency of ICI‐Q‐SF was moderate (Cronbach's *α* = 0.71), which is a limitation of the measurement tool.

Thirdly, since the data were based on only one hospital in China, demographic characteristics, cultural and lifestyle factors such as coffee consumption and smoking habits vary substantially across regions. The uneven development of prenatal services and nursing protocols in different medical institutions will also affect the application of the nomogram; prenatal care is still lacking in some regions.

Fourthly, this study only conducted internal validation and further external multi‐center validation is still needed to improve the predictive model.

Finally, due to genetic racial differences, our research may not be able to explain some results from abroad.

## 5 Conclusion

This nomogram model based on six clinical factors exhibits good discrimination, calibration, and clinical applicability for predicting stress urinary incontinence in late pregnancy. It can be used as a convenient screening tool in routine obstetric nursing. Targeted nursing interventions including pelvic floor muscle training guidance and lifestyle education are recommended for high‐risk pregnant women to reduce the incidence of stress urinary incontinence during the third trimester of pregnancy.

## Author Contributions

Conceptualization: Shanshan Shan and Yaoxiang Duan. Investigation: Junying Li, Xin Zhang, and Yu Chen. Data curation: Ying Chen and Xin Zhang, Yaoxiang Duan. Methodology: Junying Li and Hui Jiang. Formal analysis: Junying Li and Yaoxiang Duan. Funding acquisition: Shanshan Shan. Project administration: Shanshan Shan and Hui Jiang. Writing – original draft: Shanshan Shan and Ying Chen. Writing – review and editing: Hui Jiang and Yaoxiang Duan.

## Funding

This work was supported by Chinese Medical Association (Grant CMAPH‐NRP2022004).

## Ethics Statement

This study was approved by the Ethics Committee of Shanghai First Maternal and Infant Hospital, Ethics Number KS2402.

## Conflicts of Interest

The authors declare no conflicts of interest.

## Data Availability

Raw individual participant data are not publicly accessible due to patient privacy protection restrictions, but may be obtained from the corresponding author following ethical committee approval and formal data access application.
